# Impaired Right Ventricular Mechanics at Rest and During Exercise Are Associated With Exercise Capacity in Patients With Hypertrophic Cardiomyopathy

**DOI:** 10.1161/JAHA.118.011269

**Published:** 2019-02-23

**Authors:** Xiao‐Peng Wu, Yi‐Dan Li, Yi‐Dan Wang, Miao Zhang, Wei‐Wei Zhu, Qi‐Zhe Cai, Wei Jiang, Lan‐Lan Sun, Xue‐Yan Ding, Xiao‐Guang Ye, Yun‐Yun Qin, Xiu‐Zhang Lu

**Affiliations:** ^1^ Department of Echocardiography Heart Center Beijing ChaoYang Hospital Capital Medical University Beijing China

**Keywords:** exercise stress echocardiography, hypertrophic cardiomyopathy, right ventricular function, speckle‐tracking imaging, Electrophysiology, Cardiomyopathy, Hypertrophy

## Abstract

**Background:**

Impaired right ventricular (RV) function indicates RV involvement in patients with hypertrophic cardiomyopathy (HCM). We aimed to assess RV function at rest and during exercise in HCM patients and to examine the association between impaired RV mechanics and exercise capacity.

**Methods and Results:**

A total of 76 HCM patients (48 without and 28 with RV hypertrophy) and 30 age‐ and sex‐matched controls were prospectively recruited. RV function was evaluated at rest and during semisupine bicycle exercise by conventional echocardiography and 2‐dimensional speckle‐tracking imaging. Exercise capacity was measured by metabolic equivalents. RV functional reserve was calculated as the difference of functional parameters between peak exercise and rest. Compared with controls, HCM patients had significantly higher RV free wall thickness, lower RV global longitudinal strain and RV free wall longitudinal strain at rest and during exercise, and reduced RV systolic functional reserve. Compared with those with HCM without RV hypertrophy, patients with HCM with RV hypertrophy had lower metabolic equivalents. Among HCM patients, an effective correlation was seen between exercise capacity and peak exercise RV global longitudinal strain and peak exercise RV free wall longitudinal strain. A binary logistic regression model revealed several independent predictors of exercise intolerance in HCM patients, but receiver operating characteristic curve analysis indicated exercise RV global longitudinal strain had the highest area under the curve for the prediction of exercise intolerance in HCM patients.

**Conclusions:**

HCM patients have RV dysfunction and reduced contractile reserve. Exercise RV global longitudinal strain correlates with exercise capacity and can independently predict exercise intolerance. In addition, patients with HCM with RV hypertrophy exhibit more reduced exercise capacity, suggesting more severe disease and poorer prognosis.

**Clinical Perspective**

**What Is New?**

- This study evaluated the changes in right ventricular (RV) mechanics in hypertrophic cardiomyopathy (HCM) patients at rest and during exercise, and HCM patients have impaired RV mechanics and significantly reduced RV contractile reserve during exercise.
- RV dysfunction is independently associated with reduced exercise capacity in patients with HCM.
- HCM patients with RV hypertrophy exhibit a more severe reduction in exercise capacity than HCM patients without RV hypertrophy.

**What Are the Clinical Implications?**

- This study presents an enhanced understanding of the RV mechanistic characteristics in HCM patients and may have the potential to provide further evidence that RV dysfunction is associated with a disease state and poor prognosis of HCM patients.
- To optimize risk stratification of HCM patients, the importance of the evaluation of RV function needs to be emphasized in clinical practice, especially for HCM patients with RV hypertrophy.

Hypertrophic cardiomyopathy (HCM) is a genetically heterogeneous cardiomyopathy characterized by left ventricular (LV) hypertrophy with a prevalence of ≈1/500 in the general population and associated with sudden cardiac death, heart failure, atrial fibrillation, and stroke.^1^ Given the importance of LV function in the evaluation of HCM patients’ clinical status, the vast majority of previous studies performed on HCM patients have focused on the abnormality of LV structure and function but overlooked the involvement of the right ventricle, which may also be dysfunctional in HCM patients.^2^, ^3^ Indeed, as previously reported, a large number of HCM patients display right ventricular (RV) dysfunction at rest, which may result from an extension of myopathic process, sarcomere‐related protein gene mutations, augment of RV afterload, and/or shared anatomically hypertrophic interventricular septum (IVS).^4^, ^5^, ^6^ Although the precise mechanism of RV dysfunction in HCM patients remains to be explored, RV dysfunction in HCM patients may be associated with a higher risk of cardiovascular mortality.^7^ Consequently, determination of RV involvement in HCM is important for evaluation of disease status and the prognosis of HCM patients.^8^, ^9^

Impaired exercise capacity, namely, exercise intolerance, is the primary clinical feature in HCM patients and may be used to determine risk and prognosis of these patients.^10^, ^11^ Although some studies demonstrate impaired RV systolic function at rest in HCM patients,^12^, ^13^ few studies have examined RV function during exercise and explored the association of RV function and exercise capacity in HCM patients.^4^, ^14^ Exercise stress echocardiography has an important role in predicting the development of symptoms, revealing liable obstruction of the LV outflow tract, and evaluating functional capacity, heart rate (HR), and blood pressure.^15^ In addition, exercise stress echocardiography is safe and has become an essential component of the standard management of HCM, as recommended by the current guidelines defined by the European Society of Cardiology. However, given the complex anatomy of the right ventricle, accurate assessment of RV function remains a challenge. A more recently developed imaging technique, 2‐dimensional speckle‐tracking imaging (2D‐STI), allows for earlier detection of subclinical RV dysfunction and is less affected by angle dependency.^16^ Therefore, 2D‐STI is an effective tool for assessing RV function.

This study was designed to evaluate RV function in HCM patients with and without RV hypertrophy (RVH) at rest and during exercise and to determine the association between RV function and exercise capacity in these patients.

## Methods

### Study Population

A total of 93 patients with HCM who were referred to the Department of Echocardiography, Heart Center, Beijing Chao Yang Hospital, for their risk stratifications from June 2015 to January 2018 were consecutively enrolled in this study, and their RV function was assessed at rest and during exercise. These patients fulfilled the previously published HCM diagnostic criteria,^17^ which were mainly based on the echocardiographic manifestation of a maximal LV wall thickness ≥15 mm in the absence of other cardiac or systemic disease that may produce a similar degree of LV hypertrophy. In addition, HCM was also diagnosed for a patient who had a positive family history of HCM and a maximal wall thickness between 13 and 14 mm without other definite causes. Patients with 1 of the following were excluded from our study: a history of coronary artery disease with percutaneous coronary intervention and/or coronary artery bypass surgery, diabetes mellitus, New York Heart Association functional class III or IV, pulmonary hypertension, atrial fibrillation, poor imaging quality, a history of septal reduction therapy with surgical myectomy or alcohol septal ablation, significant valvular disease, or valvular prostheses. After application of exclusion criteria, patients with septal reduction therapy (n=4), coronary artery disease (n=3), valvular prostheses (n=1), and poor image quality (n=9) were excluded. Ultimately, 76 patients were included in this study. In addition, 30 age‐ and sex‐matched healthy participants were recruited as controls. The participants were divided into 3 groups: the control group, the HCM‐without‐RVH group, and the HCM‐with‐RVH group. This study was approved by the ethics committee of Beijing Chao Yang Hospital, and written informed consent was obtained from all participants.

### Echocardiography

#### Conventional measurements

Conventional echocardiography was performed on all participants to assess their cardiac function at rest and during exercise. At the standard transducer position, images were obtained with the patient at the left lateral decubitus position, using a commercially available ultrasound machine (EPIQ 7C; Philips Healthcare) equipped with an X5‐1 multiphase‐array probe. Conventional measurements were performed at rest in accordance with the current recommendations.^18^ The RV wall was observed from different echocardiographic views to identify the maximal wall thickness without including RV trabeculae. RV free wall thickness (RVWT) was measured at the end‐diastole below the tricuspid annulus at a distance approximating the length of the anterior tricuspid leaflet when it was fully open in a zoomed subcostal image with the focus on the RV midwall.^18^ If the subcostal windows were not clearly displayed, RVWT was evaluated in the left parasternal long‐axis view. A value of RVWT >5 mm was defined as the existence of RVH, as recommended.^18^ The RV basal maximal transversal dimension was measured at the end‐diastole in the RV‐focused view. Peak early and late transtricuspid filling velocities were measured at the level of tricuspid tips. Tricuspid annular plane systolic excursion was acquired in the M‐mode, and RV fractional area change was calculated as the RV area difference (diastolic–systolic) divided by the RV end‐diastolic area. RV tricuspid annular peak velocities, including systolic peak velocity of tricuspid annulus and early and late‐diastolic velocities, were acquired with pulsed tissue Doppler imaging from the lateral. LV internal diameters at the end‐diastole and end‐systole, IVS (interventricular septum), and LV posterior wall thickness were measured in the parasternal long‐axis 2D view. LV ejection fraction was calculated using the modified biplane Simpson method. LV mass was determined with the transthoracic 3D full‐volume data sets obtained from the standard apical 4‐chamber view with a frame rate >25 frames/s, and the LV mass index (LVMI) was acquired by indexing for body surface area. Peak early (shown as E) wave velocity of mitral filling and average of septal and lateral mitral annular early‐diastolic peak velocity (shown as e′) were measured, and then E/ e′ was calculated. Left atrial volume index was calculated by the maximum volume of the left atrium indexing to body surface area.

#### Deformation measurements

**Figure 1:**
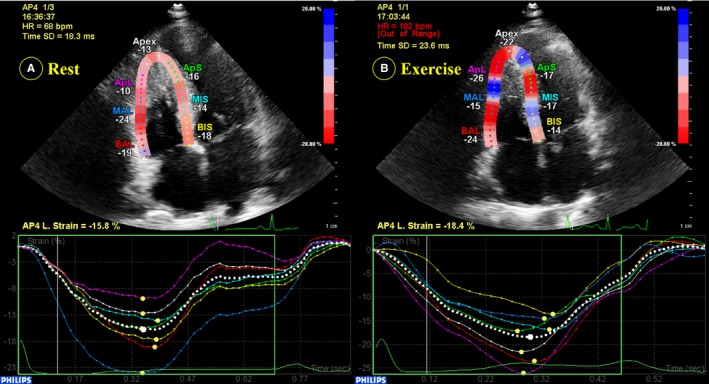
Example of right ventricular global longitudinal strain measurement in a patient with hypertrophic cardiomyopathy at rest (**A**) and during peak exercise (**B**).

The RV‐focused apical 4‐chamber view was obtained at rest and during exercise by recoding 3 consecutive heart cycles, with the frame rate optimized to 50 to 100 frames/s. 2D‐STI offline analyses were performed using the dedicated software QLAB 10.3 software (Philips Healthcare). Three RV anchor points were identified at the lateral and septal tricuspid annulus level and the RV apex by a point‐and‐click approach aiming the software to automatically track the endocardial contour. If the entire RV myocardial wall was not included in the region of interest, tracking was further adjusted manually for optimization. In addition, the pericardium was excluded in case of underestimation of measured strain. RV global and segmental strain values were obtained after software analysis (Figure 1). RV global longitudinal strain (RVGLS) was measured from RV free wall and 3 septal (basal, middle, and apical) segments, and RV free wall longitudinal strain (RVFWLS) was obtained from 3 segments of the free wall. RV contractile reserve was defined as the difference in RV strain values between peak exercise and rest.

#### Exercise stress echocardiography

All study participants underwent multistage, symptom‐limited, semisupine exercise testing using a bicycle ergometer (Ergoselect 1200, Stress Echo Couch Ergometer; Ergoline) after the resting echocardiography was performed. Workload began at 25 W and was increased by 25 W every 2 minutes. Participants were asked to maintain a rate of 55 to 60 rounds/min. The protocol required that β‐blockers or calcium channel blockers be withheld ≥24 hours before the exercise stress test. Heart rhythm, HR, and blood pressure were continuously monitored during exercise. The exercise test was promptly interrupted when a patient had 1 of the following conditions: achievement of the age‐related maximum HR, presence of significant arrhythmias, severe hypertension (blood pressure ≥240/120 mm Hg), leg muscle fatigue, or symptom intolerance such as severe chest pain, dizziness, and breathlessness. Exercise capacity was defined as metabolic equivalents (METs) and METs <7 was defined as the presence of reduced exercise capacity, as reported previously.^19^, ^20^ The following parameters were recorded at peak exercise: maximal HR, maximal systolic blood pressure, METs, and rate–pressure product (maximal HR times maximal systolic blood pressure).

### Evaluation of Reproducibility of Echocardiographic Measurements

To assess the data reproducibility, 5 patients were randomly selected from each group at least 1 month after the initial analysis, and the data sets of 15 patients were analyzed by the original investigator and a second experienced echocardiographer who were blinded to each other's measurements. Inter‐ and intraobserver variability of exercise RVGLS (RVGLS‐exe) was evaluated by intraclass correlation coefficients and the coefficient of variation.

### Statistical Analysis

Continuous variables are expressed as mean±SD and categorical variables as absolute values and percentages. Normal distribution was evaluated with the Kolmogorov–Smirnov test. Differences between groups were compared with one‐way ANOVA for normally distributed data, with the Mann–Whitney *U* test or Kruskal–Wallis test for skewed data, and with the χ^2^ test for categorical data. Correlations between different parameters were performed with the Pearson product moment correlation or Spearman rank correlation, as appropriate. Multivariable regression analysis was used to identify independent predictors of exercise intolerance in HCM patients. Receiver operating characteristic curves were used to determine the optimal cutoff values of chosen variables, and the area under the curve was used for predicting exercise intolerance. All tests were 2‐sided, and *P*<0.05 was considered statistically significant. All analyses were performed with SPSS 23.0 (IBM SPSS Statistics, v23) and MedCalc 15.6 (MedCalc Software).

## Results

### Comparison of Baseline Characteristics and Resting RV Function of Controls and HCM Patients With and Without RVH

The baseline clinical and echocardiographic characteristics of study participants at rest are summarized in Table 1. A total of 106 study participants were enrolled into this study, with 76 having HCM. Among these HCM patients, 48 did not have RVH and 28 had RVH. In addition, 11 patients were obstructive at rest and 2 patients were labile obstructive in the HCM‐without‐RVH group. Moreover, 10 obstructive and 7 labile obstructive patients were included in the HCM‐with‐RVH group. In total, 61 (80.3%) had asymmetric LV hypertrophy and 15 (19.7%) had apical hypertrophy. Three patients displayed abnormal blood pressure in response to exercise. RV intracavitary or outflow tract obstruction was not seen in all HCM patients. In addition, 69.7% and 13.2% HCM patients received β‐blockers and calcium channel blockers, respectively. No statistically significant differences were noted among control, HCM‐without‐RVH, and HCM‐with‐RVH groups regarding age, sex, and body surface area. HCM patients with or without RVH had higher LV outflow tract gradients, LVMI, IVS thickness, LV E/e′, left atrial volume index and left atrial diameter but lower resting RV strain parameters (RVGLS and RVFWLS) compared with controls (*P*<0.05 versus control). In addition, the HCM‐with‐RVH group had significantly higher resting LV outflow tract gradients, LV E/e′, IVS thickness, LVMI, and left atrial diameters compared with the HCM‐without‐RVH group (*P*<0.05 versus HCM without RVH). No significant differences in other cardiac functional parameters such as RV maximal basal transversal dimension, tricuspid annular plane systolic excursion, and RV fractional area change were observed among these groups.

**Table 1:** Comparison of Baseline Characteristics and Resting RV Function for Control Participants and HCM Patients Without and With RVH

Variables	Controls (n=30)	Patients Without RVH (n=48)	Patients With RVH (n=28)
Demographics			
Age, y	48.0±11.6	47.7±12.4	51.9±10.1
Men	23 (76.7)	39 (81.3)	22 (78.6)
BSA, m^2^	1.80±0.12	1.86±0.18	1.89±0.22
Baseline BP			
SBP‐rest, mm Hg	122.4±6.4	133.3±18.2^a^	130.7±18.6
DBP‐rest, mm Hg	75.1±5.6	79.6±11.6	84.1±13.4^b^
LV echocardiographic parameters			
LVEF‐rest, %	65.1±4.4	61.0±3.4^a^	60.2±3.6^b^
IVST, mm	8.5±0.7	16.8±4.7^a^	23.5±6.1^b^^,^ c
LVPWT, mm	8.3±0.6	11.3±2.4^a^	12.4±2.5^b^
LVIDd, mm	46.2±2.7	44.5±4.2	43.5±5.9
LVIDs, mm	25.8±3.1	23.4±4.3^a^	23.3±4.8
LVOTG‐rest, mm Hg	6.0±1.8	13.8±12.2^a^	20.0±12.6^b^^,^ c
LVOTG‐exe, mm Hg	13.1±3.3	28.9±23.0^a^	38.7±22.6^b^
LV diastolic function			
LVMI, g/m^2^	76.7±9.8	123.5±21.6^a^	150.9±29.2^b^^,^ c
LV E/e′‐rest	6.4±1.3	11.7±3.5^a^	16.0±4.3^b^^,^ c
LAVI, mL/m^2^	23.2±4.0	35.7±5.6^a^	41.3±7.7^b^
LA(ap), mm	34.1±3.1	37.9±4.8^a^	41.6±3.9^b^^,^ c
Medications			
β‐Blockers	0 (0)	33 (68.8)^a^	20 (71.4)^b^
CCB	0 (0)	4 (8.3)	6 (21.4)
Parameters of RV diameter			
RVBD, mm	34.3±2.2	33.5±2.7	34.5±3.5
RVMD, mm	22.7±2.1	22.0±3.0	21.6±3.7
RVWT, mm	4.1±0.4	4.4±0.6	6.4±0.9^b^^,^ c
Conventional RV function parameters			
TAPSE‐rest, mm	24.7±1.7	26.3±4.3	24.4±3.5
RVFAC‐rest, %	57.5±3.8	56.0±4.8	55.1±3.6
TV E/A‐rest	1.25±0.19	1.21±0.25	1.15±0.33
TD RV e′‐rest, cm/s	10.4±1.2	9.0±1.8^a^	8.0±2.0^b^
TD RV S‐rest, cm/s	14.3±2.0	12.7±2.1^a^	12.5±3.1^b^
RV E/e′‐rest	5.3±0.7	6.0±1.6	6.3±2.0^b^
PASP, mm Hg	23.0±3.5	30.9±4.0^a^	36.9±6.6^b^^,^ c
RV strain parameters			
RVGLS‐rest, %	−22.3±1.7	−17.1±2.3^a^	−13.4±2.4^b^^,^ c
RVFWLS‐rest, %	−25.2±1.2	−18.8±2.4^a^	−16.7±1.8^b^^,^ c
RVFW SRe‐rest	1.32±0.39	0.87±0.30^a^	0.81±0.27^b^

Data are expressed as mean±SD or number (percentage). BP, blood pressure; BSA indicates body surface area; CCB, calcium channel blockers; DBP, diastolic blood pressure; exe, exercise; HCM, hypertrophic cardiomyopathy; IVST, interventricular septal thickness; LA(ap), left atrial anteroposterior diameter; LAVI, left atrial volume index; LV, left ventricular; LV E/e′, ratio of early diastolic mitral flow velocity to early diastolic peak velocity of mean mitral annulus; LVEF, left ventricular ejection fraction; LVIDd, left ventricular internal diameter at end‐diastole; LVIDs, left ventricular internal diameter at end‐systole; LVMI, left ventricular mass index; LVOTG, left ventricular outflow tract gradients; LVPWT, left ventricular posterior wall thickness; PASP, pulmonary artery systolic pressure; rest, at rest; RV, right ventricular; RVBD, right ventricular maximal basal transversal dimension; RV E/e′, ratio of early diastolic tricuspid flow velocity to early diastolic peak velocity of lateral tricuspid annulus; RVFAC, right ventricular fractional area change; RVFW, right ventricular free wall; RVFWLS, right ventricular free wall longitudinal strain; RVGLS, right ventricular global longitudinal strain; RVH, right ventricular hypertrophy; RVMD, right ventricular maximal middle transversal dimension; RVWT, right ventricular wall thickness; S, systolic peak velocity of lateral tricuspid annulus; SBP, systolic blood pressure; SRe, early diastolic strain rate; TAPSE, tricuspid annular plane systolic excursion; TD, tissue Doppler imaging; TV E/A, ratio of peak early diastolic tricuspid flow velocity to peak late diastolic tricuspid flow velocity.

a *P*<0.05, HCM‐without‐RVH patients vs controls.

b *P*<0.05, HCM‐with‐RVH patients vs controls.

c *P*<0.05 HCM‐with‐RVH patients vs HCM‐without‐RVH patients.

### HCM Patients Exhibited Abnormal RV Function During Exercise

We next examined RV function in participants during exercise. As shown in Table 2, during exercise both HCM groups, without and with RVH, had lower RV free wall early diastolic strain rate and impaired RV contractile reserve, the latter of which was demonstrated by a decreased absolute value of ΔRVGLS and ΔRVFWLS compared with the control group (*P*<0.05 versus control). In addition, all HCM patients had higher E/e′ ratios and lower S and peak‐diastolic wave velocities at the lateral aspect of the tricuspid annulus during exercise compared with control participants (*P*<0.05 versus control). Furthermore, the HCM‐with‐RVH group had the smallest absolute values of RVGLS and RVFWLS among the 3 groups during exercise (*P*<0.05 versus control; *P*<0.05 versus HCM without RVH). These findings together suggest that HCM patients, regardless of whether they have RVH, exhibited dysfunctional RV function during exercise and that HCM patients with RVH had more severe RV dysfunction than HCM patients without RVH.

**Table 2:** HCM Patients Exhibited Abnormal RV Function During Exercise

Variable	Controls (n=30)	HCM Without RVH (n=48)	HCM With RVH (n=28)
Conventional RV function parameters			
TAPSE‐exe, mm	24.7±1.7	26.3±4.3	24.4±3.5
RVFAC‐exe, %	57.5±3.8	56.0±4.8	55.1±3.6
TD RV e′‐exe, cm/s	13.6±1.5	12.4±3.4^a^	10.4±2.7^b^^,^ c
TD RV S‐exe, cm/s	20.5±2.1	17.6±2.6^a^	17.1±3.7^b^
RV E/e′‐exe	5.1±0.5	6.5±1.6^a^	6.9±1.8^b^
RV strain parameters			
RVGLS‐exe, %	−26.1±1.3	−18.6±2.2^a^	−15.4±2.1^b^^,^ c
RVFWLS‐exe, %	−28.5±1.6	−20.5±2.3^a^	−18.4±1.9^b^^,^ c
RVFW SRe‐exe	1.81±0.38	1.06±0.31^a^	1.08±0.42^b^
RV contractile reserve			
▵RVGLS, %	−3.8±1.3	−1.5±0.8^a^	−2.1±1.9^b^
▵RVFWLS, %	−3.2±1.1	−1.7±1.2^a^	−1.8±0.9^b^

Data are expressed as mean±SD. E indicates early diastolic tricuspid flow velocity; e′, early diastolic peak velocity of lateral tricuspid annulus; exe, exercise; HCM, hypertrophic cardiomyopathy; RV, right ventricular; RVFAC, right ventricular fractional area change; RVFW, right ventricular free wall; RVFWLS, right ventricular free wall longitudinal strain; RVGLS, right ventricular global longitudinal strain; RVH, right ventricular hypertrophy; S, systolic peak velocity of lateral tricuspid annulus; SRe, early diastolic strain rate; TAPSE, tricuspid annular plane systolic excursion; TD, tissue Doppler imaging.

a *P*<0.05, HCM‐without‐RVH patients vs controls.

b *P*<0.05, HCM‐with‐RVH patients vs controls.

c *P*<0.05 HCM‐with‐RVH patients vs HCM‐without‐RVH patients.

### HCM Patients Exhibited More Impaired Hemodynamic Characteristics During Exercise

We next evaluated hemodynamic characteristics of these study participants during exercise. Both groups of HCM patients, with and without RVH, underwent exercise stress and did not show any severe complications (Table 3). Fatigue was the most common reason for the HCM‐without‐RVH group to discontinue the test before achieving target HR, and symptoms related to exercise intolerance like dyspnea or angina during exercise were the major reason for HCM patients with RVH to quit the test. Both groups of HCM patients, without and with RVH, showed significantly lower maximal HR and rate–pressure product (*P*<0.05 versus control) but had comparable maximal systolic and diastolic blood pressure compared with the control group during exercise. Meanwhile, when assessed by METs, both HCM groups had lower exercise capacity compared with the control group (*P*<0.05 versus control), but HCM patients with RVH had more significantly impaired exercise capacity compared with HCM patients without RVH *(P*<0.05 versus HCM without RVH), indicating that the presence of RVH in HCM patients is accompanied by enhanced hemodynamic impairment.

**Table 3:** HCM Patients Exhibited Impaired Hemodynamic Characteristics During Exercise

Variable	Controls (n=30)	HCM Without RVH (n=48)	HCM With RVH (n=28)
Peak functional parameters			
METs	8.1±1.0	6.4±1.3^a^	5.4±1.2^b^^,^ c
RPP	27027.8±2799.3	23900.1±5582.3^a^	21475.1±6470.7^b^
SBP, mm Hg	193.5±15.9	189.1±30.8	177.0±28.9
DBP, mm Hg	88.8±9.6	90.2±12.9	89.5±14.5
HR, beats/min	147.3±10.7	137.2±17.0^a^	132.2±18.7^b^
Reasons for termination			
Achieved target HR	30 (100%)	18 (37.5%)^a^	9 (32.1%)^b^
Fatigue	0 (0%)	17 (35.4%)^a^	4 (14.3%)
Symptom intolerance	0 (0%)	9 (18.8%)	13 (46.4%)^b^^,^ c
Severe hypertension	0 (%)	1 (2.1%)	0 (0%)
Frequent PVC	0 (%)	3 (6.3%)	2 (7.1%)

Data are expressed as mean±SD or number (percentage). DBP indicates diastolic blood pressure; HCM, hypertrophic cardiomyopathy; HR, heart rate; METs, metabolic equivalents; PVC, premature ventricular contraction; RVH, right ventricular hypertrophy; RPP, peak rate‐pressure product; SBP, systolic blood pressure.

a *P*<0.05, HCM‐without‐RVH patients vs controls.

b *P*<0.05, HCM‐with‐RVH patients vs controls.

c *P*<0.05 HCM‐with‐RVH patients vs HCM‐without‐RVH patients.

### Determination of the Independent Predictor of Exercise Intolerance

**Figure 2:**
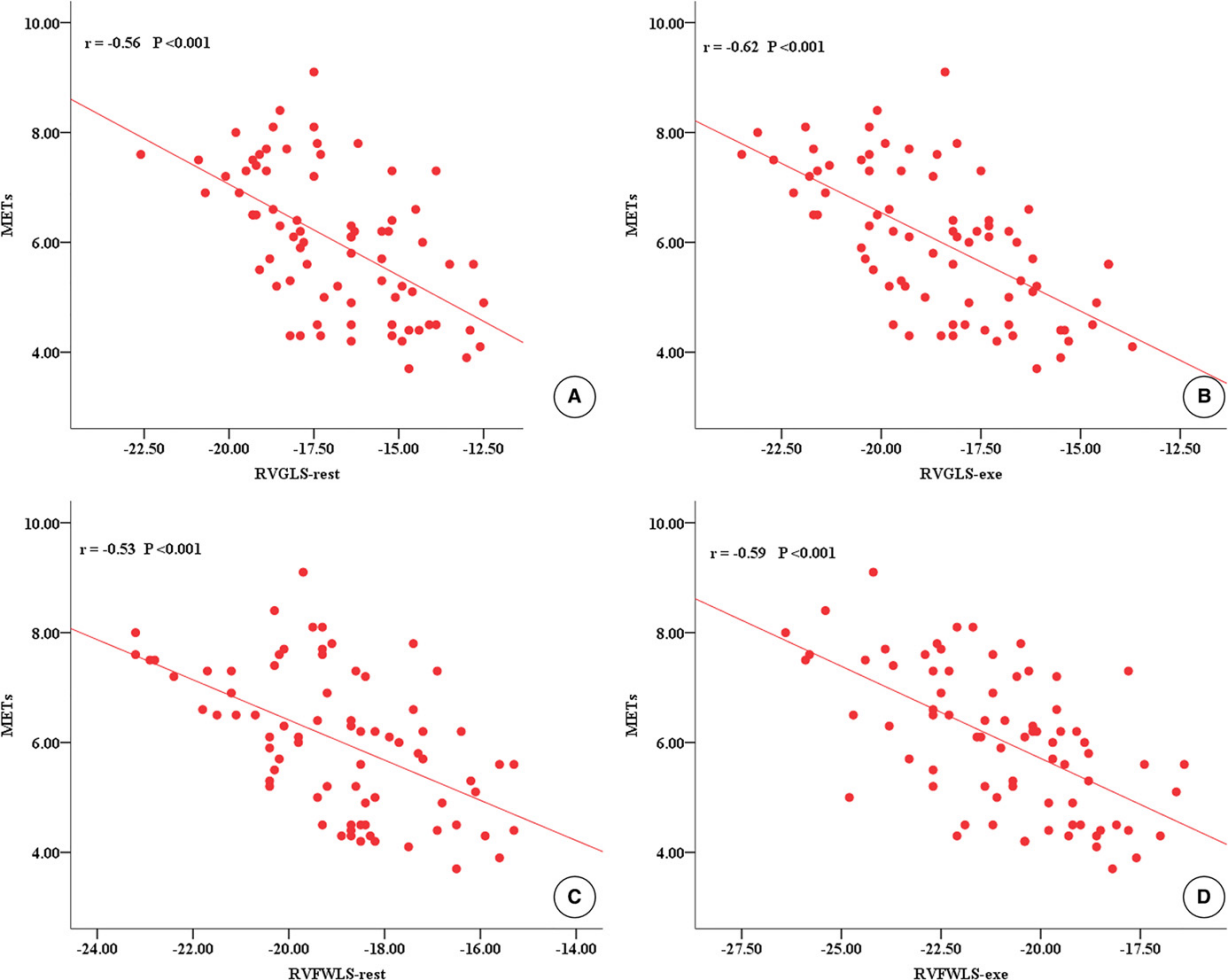
Correlations between peak METs and RVGLS‐rest (**A**), RVGLS‐exe (**B**), RVFWLS‐rest (**C**) and RVFWLS‐exe (**D**) in HCM patients. METs indicates metabolic equivalents; RVFWLS, right ventricular free wall longitudinal strain; exe, during exercise; RVGLS, right ventricular global longitudinal strain; rest, at rest.

Pearson product moment correlation or Spearman rank correlation was used to assess correlations between exercise capacity, as measured by METs and RV functional parameters. These analyses revealed moderate correlations of METs with resting RVGLS (*r*=−0.56), RVGLS‐exe (*r*=−0.62), resting RVFWLS (*r*=−0.53), and RVFWLS‐exe (*r*=−0.59) in HCM patients (*P*<0.001 for all; Figure 2). In addition, exercise capacity in HCM patients showed a moderate inverse correlation with LVMI (*r*=−0.46, *P*<0.05). Other correlations between exercise capacity and RV echocardiographic parameters at rest or during exercise are presented in Table 4.

**Table 4:** Correlations Between Exercise Capacity by Peak METs and Demographics, Echocardiographic Parameters

Variables	*r*	95% CI	*P* Value
Age, y	−0.40	−0.58 to −0.20	<0.001
Peak HR, b/m	0.48	0.29–0.64	<0.001
Peak SBP, mm Hg	0.43	0.23–0.60	<0.001
Peak DBP, mm Hg	0.18	−0.05 to 0.39	0.121
LVOTG‐exe, mm Hg	−0.39	−0.57 to −0.18	<0.001
LVOTG‐rest, mm Hg	−0.47	−0.63 to −0.28	<0.001
LVE/e′‐rest	−0.26	−0.46 to −0.04	0.021
LAVI, mL/m^2^	−0.38	−0.56 to −0.17	<0.001
LVMI, g/m^2^	−0.46	−0.62 to −0.27	<0.001
IVST, mm	−0.27	−0.47 to −0.05	0.018
RVWT, mm	−0.37	−0.55 to −0.16	0.001
RVFAC‐exe, %	0.26	0.04–0.46	0.022
TAPSE‐exe, mm	0.45	0.24–0.61	<0.001
TV E/e′‐exe	−0.35	−0.53 to −0.13	0.002
RVGLS‐rest, %	−0.56	−0.70 to −0.39	<0.001
RVGLS‐exe, %	−0.62	−0.74 to −0.46	<0.001
RVFWLS‐rest, %	−0.53	−0.68 to −0.35	<0.001
RVFWLS‐exe, %	−0.59	−0.72 to −0.42	<0.001

DBP indicates diastolic blood pressure; exe, exercise; HR, heart rate; IVST, interventricular septal thickness; LAVI, left atrial volume index; LV, left ventricular; LVE/e′, ratio of early diastolic mitral flow velocity to early diastolic peak velocity of mean mitral annulus; LVMI, left ventricular mass index; LVOTG, left ventricular outflow tract gradients; METs, metabolic equivalents; rest, at rest; RVFAC, right ventricular fractional area change; RVFWLS, right ventricular free wall longitudinal strain; RVGLS, right ventricular global longitudinal strain; RVWT, right ventricular wall thickness; SBP, systolic blood pressure; TAPSE, tricuspid annular plane systolic excursion; TV E/e′, ratio of early diastolic tricuspid flow velocity to early diastolic peak velocity of lateral tricuspid annulus.

**Figure 3:**
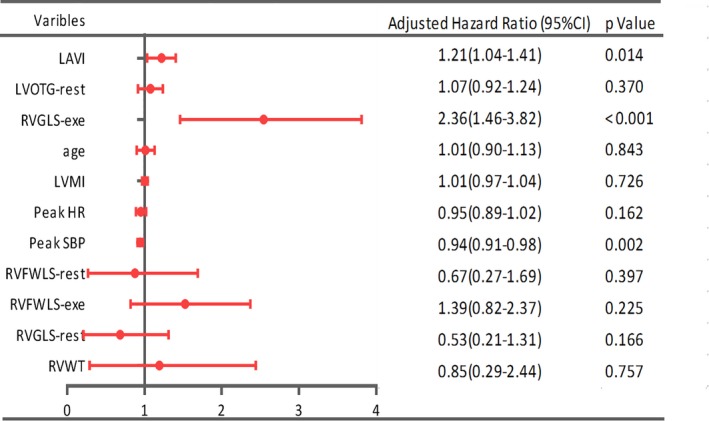
Logistic regression was performed to identify the right ventricular functional parameters independently associated with exercise capacity. exe indicates exercise; HR, heart rate; LAVI, left atrial volume index; LVMI, left ventricular mass index; LVOTG, left ventricular outflow tract gradients; RVFWLS, right ventricular free wall longitudinal strain; RVGLS, right ventricular global longitudinal strain; RVWT, right ventricular wall thickness; SBP, systolic blood pressure.

We next used univariate regression analyses to assess the association between exercise intolerance (METs <7) and RV functional parameters, LVMI, and clinical characteristics, and parameters with significant correlations identified by this approach were incorporated into a multivariable logistic regression model to identify independent predictors of exercise intolerance. This analysis revealed that RVGLS‐exe was the independent predictor of exercise intolerance (Figure 3).

### Identification of Parameters Associated With Exercise Intolerance

**Figure 4:**
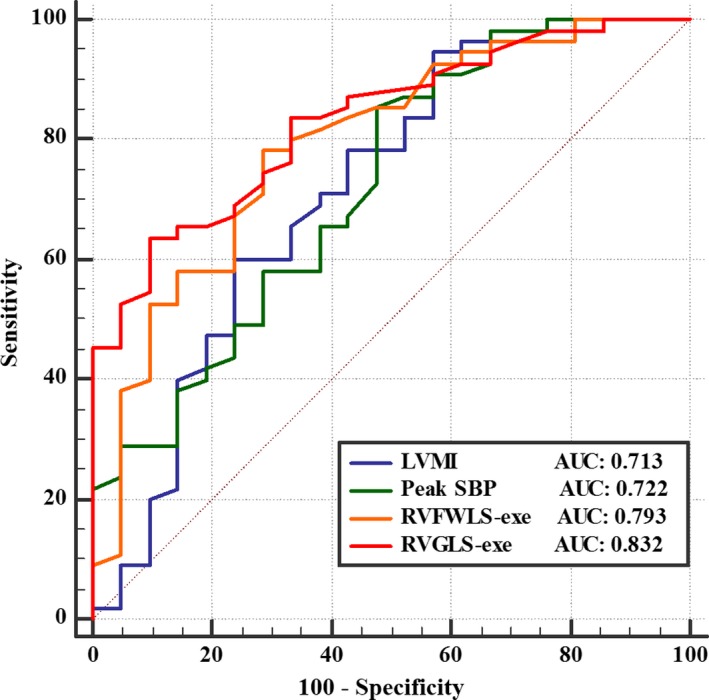
Receiver operating characteristic curves of the accuracy of RVGLS‐exe and other parameters to identify hypertrophic cardiomyopathy patients with exercise intolerance. AUC indicates area under the curve; LVMI, left ventricular mass index; RVFWLS, right ventricular free wall longitudinal strain; RVGLS, right ventricular global longitudinal strain; exe, exercise; SBP, systolic blood pressure.

Receiver operating characteristic analysis (Figure 4) revealed that RVGLS‐exe had better capability to identify patients with exercise intolerance compared with other parameters in HCM patients, with an area under the curve of 0.832 (*P*<0.05), sensitivity of 63.6%, and specificity of 90.5% (Table 5).

**Table 5:** Determination of Cutoff Values of Parameters for Prediction of Exercise Intolerance by Receiver Operating Characteristic Curve Analysis

Variables	AUC	95% CI	Cut off	Sensitivity (%)	Specificity (%)
LVMI, g/m^2^	0.713	0.597–0.811	105.5	94.6	42.9
Peak SBP, mm Hg	0.722	0.607–0.819	205	85.2	52.4
RVFWLS‐exe, %	0.793	0.684–0.877	−21.7	78.2	71.4
RVGLS‐exe, %	0.832	0.729–0.908	−18.4	63.6	90.5

AUC indicates area under the curve; exe, exercise; LVMI, left ventricular mass index; RVFWLS, right ventricular free wall longitudinal strain; RVGLS, right ventricular global longitudinal strain; SBP, systolic blood pressure.

### Determination of Reproducibility

Inter‐ and intraobserver variability of RVGLS‐exe was presented by intraclass correlation coefficients and the coefficients of variation in the subjects randomly selected from each group (n=15). Intraclass correlation coefficients of inter‐ and intraobserver variability were 0.81 and 0.86, respectively, for RVGLS‐exe. The coefficients of variation for inter‐ and intraobserver variability were −5.1±1.2% and −4.8±1.1%, respectively, for RVGLS‐exe. These observations document high reproducibility of the measurements obtained in our study.

## Discussion

To the best of our knowledge, this study is the first to assess the relationship of RV function and its contractile reserve with exercise capacity in HCM patients with or without RVH, both at rest and during exercise, in the same experimental setting. We had 4 main findings. First, RV myocardial function was impaired in HCM patients at rest and during exercise, and RV contractile reserve was reduced during peak exercise, regardless of whether HCM patients had concomitant RVH. Second, HCM patients with RVH had lower RVGLS, RVFWLS, and exercise capacity compared with HCM patients without RVH and controls. Third, RV wall thickness, RVGLS, and RVFWLS at rest and during exercise were associated with exercise capacity. Fourth, RVGLS‐exe had the strongest correlation with exercise capacity, even after adjustment for other variables, and was the most powerful predictor for identifying HCM patients with reduced exercise capacity compared with other parameters. Our findings provide a significant reference for the clinical management of HCM patients.

### Development of RVH in HCM Patients and Potential Mechanisms

Although HCM is a genetically heterogeneous cardiomyopathy characterized by LV hypertrophy, a number of studies have shown abnormal RV morphology and function in HCM patients that manifests mainly as RV remodeling and RV systolic and diastolic dysfunction.^12^, ^21^, ^22^ A more recent study reported that 53% of HCM patients exhibited RVH.^2^ In the present study, we found not only that HCM patients had abnormal RV function during exercise but also that 28 of 76 HCM patients had RVH; therefore, our findings further support the notion that HCM patients commonly develop RVH. The presence of RVH may further increase stiffness and decrease compliance of the ventricular wall, which in turn leads to increased RV filling pressure and heightened diastolic dysfunction, as demonstrated by the increased risk of heart failure, malignant arrhythmia, thromboembolism, and sudden cardiac death in HCM patients with RVH.^9^ Thus, concomitant RVH in HCM patients may indicate poor prognosis. Consequently, prevention or reduction of the occurrence of RVH in HCM patients may improve the clinical outcomes of HCM patients after treatment.

Although the exact mechanisms underlying the development of RVH in HCM patients are not clear, multiple factors have been proposed to play a role. A mutation on the *MYBPC3* (myosin binding protein C, cardiac) gene, for example, was found to be linked to the reduced synthesis and abnormal assembly of sarcomere‐associated proteins, and this affected the phenotypic expression of HCM.^23^ Increased calcium sensitivity, which correlates to myocardial hypercontractility, was also shown to contribute to the pathogenesis of RVH.^24^ In addition, depletion of myocardial energy, which is fundamental for effective cardiac muscle contraction and efficient cardiac output, was reported to regulate RV remodeling.^25^ Further understanding the mechanisms that promote the pathogenesis of RVH in HCM patients will facilitate the design of strategies aimed at diminishing adverse outcomes and improving the quality of life of HCM patients.

### RV Diastolic and Systolic Dysfunction in HCM Patients

As mentioned, >50% of HCM patients had RV diastolic and systolic dysfunction. The E/e′ ratio is a reliable parameter and has been recommended by the current guideline for evaluating RV diastolic function.^18^ In the present study, we also found that the RV filling pattern was significantly more restrictive in HCM patients, both with and without RVH, than in controls, as demonstrated by a higher E/e′ ratio in patient groups. Consistent with those previous studies, our observations suggest impairment in RV diastolic function in HCM patients, regardless of the presence of RVH. Because RV diastolic dysfunction has been considered an independent predictor of adverse outcome in patients with HCM, the risk of death from heart failure for HCM patients with RV diastolic dysfunction and an increased E/e′ value is increased by 1.6 times.^26^ It will be interesting to examine the prognostic value of RV diastolic function in these patients for a longer period of time.

Recently, 2D‐STI, which can provide early detection of subclinical myocardial dysfunction,^27^ has offered a sensitive method for the study of RV myocardial mechanics at rest and during exercise. In this study, we used 2D‐STI to appraise RV systolic function based on RV strain parameters (RVGLS, RVFWLS) and showed impaired RV function in patients with HCM. However, we did not observe any significant differences in conventional echocardiographic parameters such as tricuspid annular plane systolic excursion and RV fractional area change between HCM patients and controls, which was in agreement with findings from a previous study.^28^ Several mechanisms have been proposed to be linked to the pathogenesis of RV dysfunction in HCM patients. First, myocardial disarray and interstitial fibrosis may contribute to the progression of RV diastolic or systolic dysfunction. A study based on cardiac magnetic resonance has shown that late gadolinium enhancement, a manifestation of replacement fibrosis, was associated with abnormal RV mechanics.^3^ Microcirculatory ischemia,^29^ which is mainly attributed to abnormal intramural arteries characterized by intimal proliferation and luminal narrowing, was also correlated with RV dysfunction. It is well established that the RV wall is composed of superficial layer fibers that are aligned circumferentially and deep layer fibers arranged longitudinally, the latter of which are most vulnerable to ischemia. In addition, energy deficits also contribute to the pathogenesis of RV systolic dysfunction.^30^

### Correlation Between RV Function and Exercise Capacity

RV dysfunction is an important cause of exercise intolerance, which is an independent predictor of adverse outcomes in patients with HCM.^31^, ^32^ Exercise stress echocardiography has been proposed to assess exercise capacity in symptomatic HCM patients who failed to induce LV outflow tract obstruction ≥50 mm Hg by bedside maneuvers, according to the current guideline.^17^ In our study, HCM patients either with or without RVH had higher RV wall thickness and tricuspid E/e′ both at rest and at peak exercise compared with controls, indicating the presence of RV remodeling and increased RV filling pressure. These abnormal RV functions may contribute to reduced exercise capacity. Conversely, we showed that increased IVS thickness and LVMI were also correlated with reduced exercise capacity, as measured by METs. Because of the close correlation of exercise capacity with age and sex, we further analyzed these correlations in the multivariable regression model. We found that it was not LVMI; rather, maximal systolic blood pressure during exercise and RVGLS‐exe were independent factors affecting exercise intolerance, suggesting that HCM patients with an abnormal BP response to exercise, which was demonstrated as a risk factor of sudden cardiac death in HCM patients,^15^ exhibit reduced exercise capacity.

RV strain values were sensitive markers of exercise capacity.^31^ Indeed, in the present investigation, we found that RVFWLS and RVGLS, either at rest or during exercise, were strongly correlated with exercise capacity assessed by METs in HCM patients. Moreover, RVGLS‐exe was an independent predictor of exercise intolerance. We also showed that RV strain absolute values were significantly lower in patients with HCM than in controls, further supporting the existence of RV subclinical myocardial dysfunction in HCM patients, as reported by others.^14^, ^28^ Although global RV systolic function comprises 3 functional parts—RV longitudinal systolic function, RV radial function, and anteroposterior systolic function^33^, ^34^—80% of the total stroke volume was shown to be dependent on shortening of the longitudinal axis.^33^, ^35^ Consequently, we hypothesize that impaired exercise capacity in HCM patients may result from decreased RV longitudinal strain due to subclinical myocardial dysfunction, leading to improper increase in RV contractile reserve during exercise according to the Frank–Starling law. In addition, we found that RVGLS‐exe had the highest area under the curve for predicting exercise intolerance among all echocardiographic variables and that an RVGLS‐exe cutoff value of −18.4% identified exercise intolerance in HCM patients with 63.6% sensitivity and 90.5% specificity. Moreover, RVGLS was more reliable in predicting reduced exercise capacity in HCM patients compared with other predictors such as RVFWLS. Mechanistically, because of hypertrophic IVS, the amplitude of ventricular septum bulging into the right ventricle was reduced, and the force of stretching the RV free wall over the septum was weakened during systole, both of which impaired RV pump function. Because a reduction in exercise capacity was an independent predictor of poor prognosis in HCM patients,^31^ our identification of RVGLS‐exe measured by 2D‐STI as an evaluator of exercise capacity points to the potential application of RVGLS‐exe in the risk stratification and prognostic assessment of HCM patients.

### Clinical Implications

Our findings present a novel understanding of HCM characteristics. RV systolic or diastolic dysfunction may increase risk of cardiovascular adverse events in HCM patients and indicates poor prognosis, as demonstrated by previous studies.^2^, ^26^ In clinical practice, we emphasize the importance of the evaluation of RV function in patients with HCM and the rapid identification of patients with RV dysfunction, with the goal of eventually reducing the incidence of clinical adverse events, especially in HCM patients with RVH.

### Limitations

Several limitations of this study should be acknowledged. First, it was a single‐center observational study with a relatively small sample size; therefore, the inherent sampling bias of this kind study could not be precluded. Second, LV‐specific software was used to analyze RV function because no 2D‐STI dedicated software was available for RV deformation measurements during the course of this study. Third, as mentioned, global RV systolic function has 3 functional parts, but we evaluated only RV longitudinal systolic function because it contributed to 80% of the RV total stroke volume. Nevertheless, we cannot rule out the possibility that the other 2 RV functions may be compromised in HCM patients with and/or without RVH, and this may need to be explored further. Fourth, the gold standard for imaging evaluation of RV structure and function is cardiac magnetic resonance, which was not performed in our study given its time‐consuming nature and high cost. Fifth, in this study, we mainly evaluated the correlation between RV function and exercise capacity and did not examine the effect of LV function on exercise capacity in patients with HCM. Finally, the predictive value of RV strain parameters at peak exercise for predicting the prognosis of HCM patients needs to be further confirmed by future multicenter prospective studies.

## Conclusions

In this study, we demonstrated that patients with HCM have reduced RV systolic and diastolic function at rest and at peak exercise and have significantly impaired RV systolic function reserve during exercise. In addition, RV function is associated with exercise capacity in HCM patients, and HCM patients with RVH exhibit more severe reductions in exercise capacity than HCM patients without RVH. RVGLS‐exe is an independent predictor of exercise intolerance in HCM patients, indicating that it may be used for risk stratification and prognostic assessment of HCM patients.

## Author Contribution

Lu, Li, Wu and Wang designed the study. Wu, Zhang, Zhu, Cai, Jiang, Sun, Ding, Ye and Qin were involved with the inclusion of study participants and acquisition of data. Lu, Li, Wu and Zhang analyzed the data. Lu and Wu drafted and wrote the article. Lu, Li, Wu, Cai and Sun revised the article critically for intellectual content. All authors gave intellectual input to the study and approved the final version of the article.

## Sources of Funding

This work was supported by the Natural Science Foundation of China (NSFC No. 81571683), Beijing Chao‐Yang Hospital 1351 Talent Training Plan (No. CYMY‐2017‐28).

## Disclosures

None.

## Data Availability

The data that support the findings of this study are available from the corresponding author on reasonable request.
